# Method to determine instantaneous transient responses in pressurized pipes from transfer functions and state space for evaluation of leak signals

**DOI:** 10.1016/j.mex.2024.102762

**Published:** 2024-05-14

**Authors:** Edgar Orlando Ladino-Moreno, César Augusto García-Ubaque, Oscar Gabriel Espejo-Mojica

**Affiliations:** Universidad Distrital Francisco José de Caldas, Bogotá, Colombia

**Keywords:** Esp8266, Iot, Leaks, System dynamics, Transfer function, Transients, Water Hammer, Method to determine instantaneous transient responses in pressurized pipes

## Abstract

This article addresses the impact of transient pressure anomalies in hydraulic systems, triggered by the opening or closing of valves or pumps, instantly disturbing the line of hydraulic gradient (LGH). This variation in pressure has significant consequences both in hydraulic and structural terms for water networks. Most of the existing techniques to detect transients in water distribution systems use asynchronous methods, generating timeless information that limits the response capacity in critical situations. Therefore, an automatic transient detection system based on the Internet of Things (IoT) is proposed, capable of identifying overpressure or underpressure pulses in soft real-time, activating alarms to facilitate decision-making. This approach helps maintain the safety of the water distribution system and prevent leaks in the network. Furthermore, a model of the transient behavior of pressure and flow is presented by linearizing the water hammer equations from the Laplace transform, thus generating a transfer function that describes the algebraic relationship between the outlet and inlet of the hydraulic system.

• The transient analysis of the hydraulic system prototype underscores its high sensitivity to initial conditions, attributed to turbulence. This observation suggests the possible presence of a dynamic strange attractor related to water hammer phenomena in pressure pipes.
• The methodology involving transfer functions and state-space models enables the assessment of how leaks impact the transient responses of the system, including the magnitude, duration, and frequency of disturbances generated by them.
• The proposed method introduces a dynamic transfer function capable of identifying instantaneous changes over time in terms of flow and pressure.

## Graphical abstract

**Figure:**
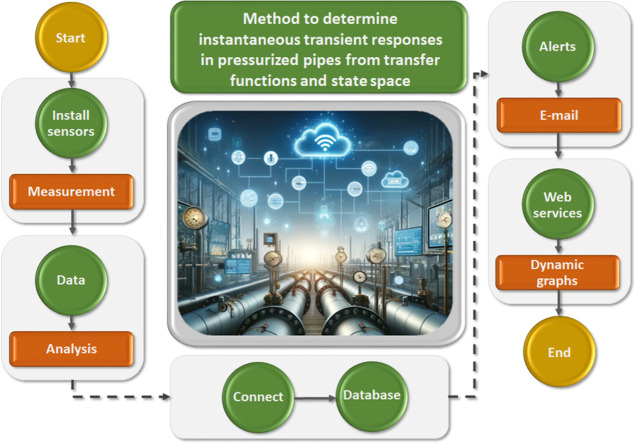

Specifications table

**Table:** 

Subject area:	Engineering
More specific subject area:	Hydraulic
Name of your method:	Method to determine instantaneous transient responses in pressurized pipes
Name and reference of original method:	N/A
Resource availability	https://www.edgarladino.com/vibraciones-iot-tuber%C3%ADas

## Background

In the field of safety of hydraulic systems intended for water distribution, this article exhaustively addresses the challenge posed by transient pressure anomalies, caused by the opening and closing of valves or pumps, leaks, and illegal connections. Instantaneous disturbance at the line of hydraulic gradient (LGH) can have significant hydraulic and structural consequences on water networks. The main motivation behind our methodology lies in addressing the limitations of the techniques that are currently presented, producing asynchronous information and a lack of network monitoring, reducing the response capacity in critical situations. We propose an innovative Internet of Things (IoT)-based approach for automated real-time transient detection. This system not only identifies overpressure or underpressure pulses but also activates immediate alarms, providing a valuable tool for real-time decision-making. The article presents a model of the transient behavior of pressure and flow, using the Laplace transform to linearize the water hammer equations. This approach makes it easier to understand the algebraic relationship between the input and output of the hydraulic system. The high sensitivity of the prototype of the hydraulic system to the initial conditions, linked to turbulence, is highlighted, so the presence of a dynamic strange attractor associated with water hammer phenomena in pressure pipes is possible. The proposed methodology, which includes transfer functions and state space models, allows a comprehensive evaluation of how leakage affects the system's transient responses. Furthermore, this is supported by the introduction of a dynamic transfer function, capable of identifying instantaneous changes over time. Thus, the importance of this study lies in its comprehensive approach, which combines the development of an automatic system for transient detection based on the Internet of Things (IoT) with the implementation of advanced transfer functions. This system allows establishing early warnings that facilitate the adoption of contingent measures in the event of sudden transients in the hydraulic network. This response capacity contributes significantly to improving the safety of the hydraulic system, allowing timely interventions that prevent damage to the system's infrastructure.

## Method details

The smart monitoring of water distribution systems serves to prevent transients and to locate and quantify leaks in the system. Thus, a soft real-time measurement system is crucial for measuring the flow behavior and the network pressure, as well as for preventing leaks and cracks in the pipeline system. These methodologies contribute to the intelligent management of water from the Internet of Things (IoT) and artificial intelligence (AI) establishing an Intelligent Water Management System. Therefore, the water sector must adopt the technological changes that are currently associated with the implementation of IoT for monitoring hydraulic systems [1] In fact, it is possible to manage the water distribution network with IoT sensors [2] which is useful for monitoring and automation [3,4] states that emerging technologies related to wireless sensor networks are viable for monitoring water distribution systems. Similarly, [5]proposed an intelligent system based on IoT for leak detection and implemented the nano microcontroller (^Ⓡ^Arduino) and the flowmeter (YF-S201) to obtain the flow of water inside the pipe in l/hr. from the generation of pulses with an accuracy of +/−10 %. The captured data was sent to the server. The relationship between flow velocity difference and leakage was found to be nearly linear. Therefore, they proposed a web interface to display the flow rate difference of the system. In addition, [6]proposes an integral system based on intelligent sensors and actuators with Big Data processing for monitoring the structural and hydraulic status of oil pipelines. The data produced by the sensor due to the vibration of the wave in the hydraulic system determines if the flow behaves in a stable or unstable way [7]

[8] he argues that implementing smart sensors and digital IoT systems to monitor pumping systems in soft real-time can predict potential failures. Similarly, the use of water flow sensors and microcontrollers contributes to real-time monitoring for leak detection by generating pipe flow rates in system indicators [9] The IoT capacity is focused on the detection, analysis, and processing of data associated with the inference of variables of urban environments [10] The use of the Internet of Things (IoT) and Information and Communication Technologies (ICT) can contribute to the conservation of water resources. In the context of smart cities, IoT is used to improve the efficiency and performance of urban infrastructure [11]. The importance of real-time monitoring of the different variables involved in Civil Engineering projects lies in the need to know the real-time variation of the observed variable or phenomenon that is associated with changes generated by on-site conditions. For example, real-time monitoring of the depth of rivers establishes an early warning mechanism for flood risks in populated areas. As a result, an information system synchronized with the behavior of the water event, the emergence of methodologies associated with risk management, and the social appropriation of knowledge are obtained. Likewise, the synchronous monitoring of structures, buildings, slopes, transit and transportation, water distribution systems, and sanitation systems leads to the structuring of smart cities based on IoT and AI. In environmental terms, IoT establishes precise control over water resources data, identifying the main players in the water sector; in this way, proactive innovation is achieved, and different problems associated with water scarcity might be solved [12] The implementation of technologies such as IoT and AI establish the progressive transformation of monitoring, storage, control, and automation of water distribution systems [13] The materialization of a water distribution network system monitored in soft real-time through IoT leads to the identification of possible leaks, the visualization of hydraulic system patterns, and the location of erroneous and fraudulent connections generated by illegal connections. Given the above, it is possible to configure a synchronous alarm system in the event of anomalies caused by the variation of flow and pressure in the network. IoT represents a powerful tool for real-time identification of pressure variation, articulated to artificial intelligence algorithms that classify areas of possible water mass leaks, which is particularly useful for leak detection. However, this ability to locate leaks depends directly on the location of the sensors within the network, which is why it is necessary to study the behavior of leaks in the hydraulic sector based on expert knowledge. Thus, a binomial is established between the metaheuristic models and the knowledge of the operators of the water distribution system. This produces the integration of IoT, AI, and expert knowledge to produce a combinatorial optimization model for the intelligent management of water resources for the structuring of smart water networks. The identification and location of overpressures produced by transients in the water distribution network from an IoT scheme have two main uses. On the one hand, the development of contingency plans for eventual explosions in main conduction pipes. Therefore, industrial machinery and IoT software can be integrated, enabling users to access this information in soft real-time via a web interface [14]. On the other hand, the strategic location of pressure regulating valves strategically dampens the transients generated in the network and the compensation chambers in the case of hydroelectric power plants. In this way, the water moves through the pressure pipe and is diverted when the valves in the chamber's spout are closed, thus the destructive pressure energy is transformed into potential energy. This strategic location of sensors and regulating valves in water distribution systems constitute an optimization problem that can be solved using graph theory exploration algorithms, articulated with expert knowledge of the network. For example, [15] adopted algorithms from graph theory to suggest the location of sensors in water transport systems. The proposed approach provides decision support for public water supply companies. Also, [16] proposed an IoT-based system to locate damage in oil pipelines from pressure pulses based on the principle of pipe vibration. These data were sent to the ^Ⓡ^ThingSpeak server with a 15-second delay. The overpressures generated in the pressurized hydraulic systems generated collateral effects in structural terms of the pipe network, affecting the water supply. Inadequate handling of transients produced in the water conduction network creates a high risk in terms of hydraulic explosions for larger-diameter main lines. This condition implies the development of mechanisms and the adoption of methodologies that minimize the latent risk of explosion in the system due to overpressure in some weak point of the pipeline [17] Transients can introduce large pressure forces and instantaneous increases in accelerations in water distribution systems. These disturbances can cause failures in pumping systems and hydraulic devices, fatigue of the pipe material, and ruptures [18] Therefore, the importance of implementing monitoring systems based on IoT is evident to identify the transients generated in the water supply network and thus reduce the risks and side effects of a possible hydraulic explosion. This study aims to propose a new prototype for monitoring transients in soft real-time. The proposed experimental system can detect transients in soft real-time and issue an alarm via Twitter or email. It was demonstrated that it is possible to generate early warning systems based on IoT for the control and damping of transients produced by the hydraulic network. For this reason, the importance of considering the incorporation of the development of IoT systems in the contents of the professional programs of educational institutions [19].

### Transients in pressure pipes

The hydraulic theory for the study of water hammer proposes two models associated with the pipe material and the flow density. The first hypothesis is related to the rigid column model in which the pipe does not experience structural changes (inelastic) due to the transient generated by the abrupt closure of the downstream valve; in this case, the flow presents a constant density throughout the entire system. The second model establishes that the conduction pipe experiences variation in elastic terms of the material that conducts the fluid in the event of an overpressure or under-pressure event generated by a water hammer; in this case, the flow is compressible. The latter constitutes the best approximation in theoretical terms to the real and experimental behavior of the transient phenomenon. It has been shown that the pipe material is incapable of rapidly dissipating the wave event under the action of a transient; therefore, water distribution systems cannot attenuate ripples caused by transmission effects [20] For a hydraulic system in which an abrupt closure of a downstream valve is generated, a pressure wave is produced that affects the behavior of the Hydraulic Gradient Line (HGL). Then the variation of HGL depends on the position x along the pipe at time t. This behavior establishes a set of nonlinear partial differential equations to determine the velocity and piezometric head along the pipe. From the principle of conservation of mass, for an unsteady flow we have

(1) $$\frac{\partial H}{\partial t}+V\frac{\partial H}{\partial x}+\frac{a^{2}}{g}\frac{\partial V}{\partial x}-Vsen\left(\alpha \right)=0$$

where: a: pressure wave speed (m/s); V: average velocity in the pipe, parallel to the x axis (m/s); H: hydraulic gradient line (m); x: distance along the pipe (m). The solution of nonlinear differential equations is possible using the method of characteristics (MOC) and the finite difference method [21] If the velocity perpendicular to the tube axis is reduced to zero, a one-dimensional system defined by

(2) $$\frac{\partial V}{\partial t}+V\frac{\partial V}{\partial x}+g\frac{\partial H}{\partial x}+f\frac{V\left|V\right|}{2D}=0$$

where: D: inner diameter of the pipe (m);f: coefficient of friction from the Darcy–Weisbach equation. The Partial Differential Eqs. (1) and (2) can be solved using the method of characteristics (MOC). This method determines the values of the load H and the velocity V from regular intervals of position x and time t [22] thus

(3) $$C^{+}=\frac{\partial V}{\partial t}+\frac{g}{a}\frac{\partial H}{\partial t}+f\frac{V\left|V\right|}{2D}=0;\;\frac{\partial x}{\partial t}=+a$$

(4) $$C^{-}=\frac{\partial V}{\partial t}-\frac{g}{a}\frac{\partial H}{\partial t}+f\frac{V\left|V\right|}{2D}=0;\;\frac{\partial x}{\partial t}=-a$$

The MOC aims to find the value of the flow and pressure from a mesh concerning the length of the pipe and the initial hydraulic head; thus, it generates signals in space-time as lines that propagate to the right $\left(C^{+}\right)$ and to the left $\left(C^{-}\right)$.

(5) $$C^{+}=H_{i,j+1}-H_{i-1,j}+\frac{a}{gA}\left(Q_{i,j+1}-Q_{i-1,j}\right)+\frac{f\Delta x}{2gA^{2}D}\left(Q_{i-1,j}\left|Q_{i-1,j}\right|\right)=0$$

(6) $$C^{-}=H_{i,j+1}-H_{i+1,j}-\frac{a}{gA}\left(Q_{i,j+1}-Q_{i+1,j}\right)-\frac{f\Delta x}{2gA^{2}D}\left(Q_{i+1,j}\left|Q_{i+1,j}\right|\right)=0$$

For an experimental simulation scenario of a water hammer, a long pipeline can present the same behavior in the event of a transient event as a short pipe with a reduced closing time.

(7) $$T<\frac{2L}{c},quick\;closing\to a;L>\frac{cT}{2}long\;conduction$$

(8) $$T\;<\;\frac{2L}{c},\;slow\;closing\;\to a;\;L\;<\;\frac{cT}{2}\;slow\;conduction$$

The speed of the wave in pipes is defined by the Joukowski formula

(9) $$c=\frac{\sqrt{\frac{k}{\rho }}}{\sqrt{1+\frac{kD}{E\varepsilon }}}$$

where: k: fluid module of elasticity; ρ: fluid density; D: pipe diameter (m); E: module of elasticity of the material constituting the pipe; ε: pipe thickness. For the case of water, the speed of the pressure wave in the pipe is calculated as follows

(10) $$a=\frac{9.900}{\sqrt{47.6+C\frac{D}{\varepsilon }}}$$

where: C: coefficient depending on pipe material; D: inner diameter of pipe (m); ε: rugosity (m). For inelastic systems, it is feasible to establish the hydrodynamics of the flow from Newton's second law of motion as follows

(11) $$\partial H=f\frac{L}{D}\frac{V\left|V\right|}{2g}+\frac{L}{g}\frac{\partial V}{\partial t}$$

where: ∂H: hydraulic gradient line change (m). For stationary states, the change of velocity in Eq. (11) is equal to zero and the calculation of the head loss due to friction reduces to the Darcy-Weisbach equation.

## Experimental hydraulic design

**Fig. 1:**
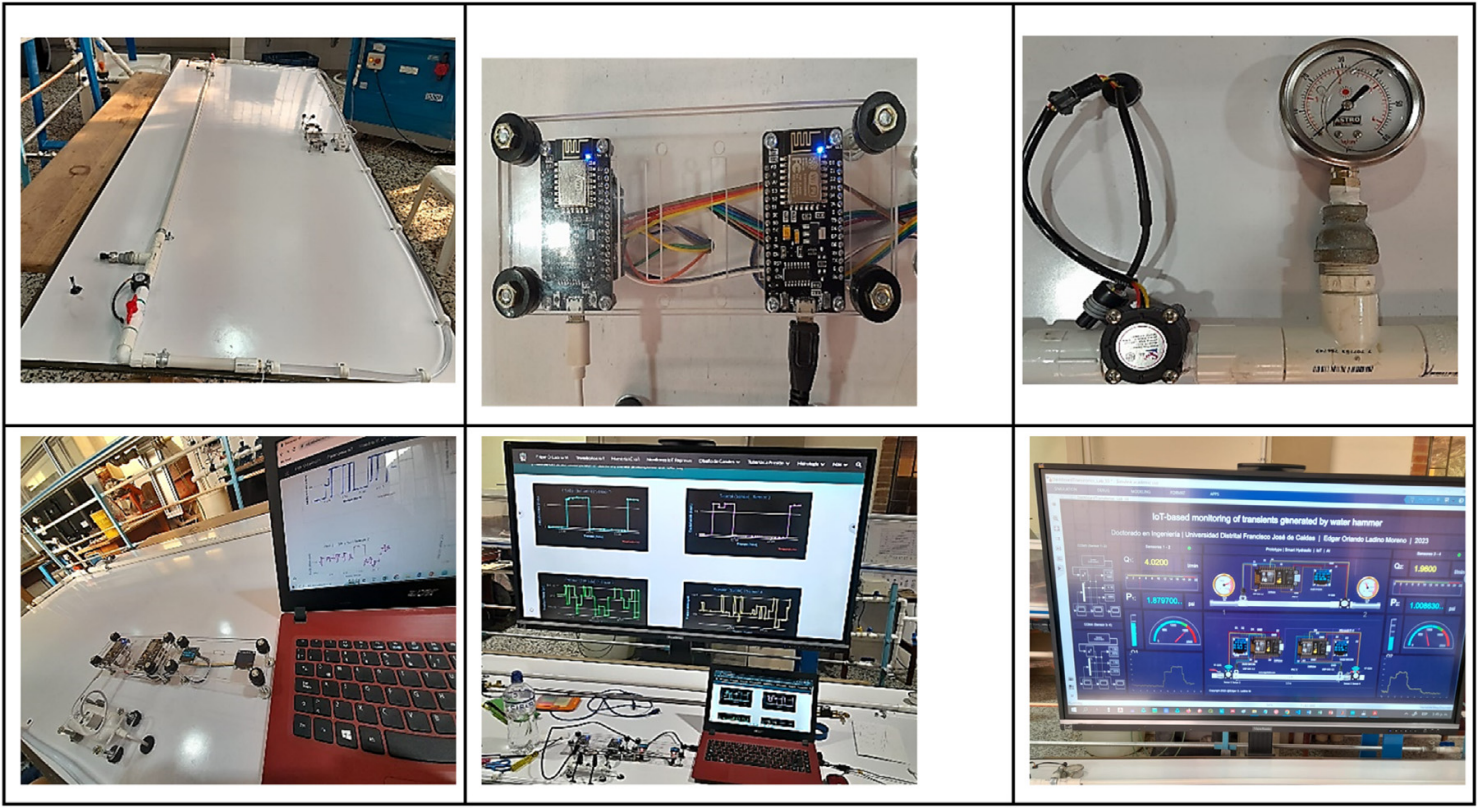
Prototype (Hydraulics Laboratory, Universidad Distrital Francisco José de Caldas).

The system comprises a 1.5 m PVC ½" pipe, two ½" PVC valves, and two ½" PVC unions. It is powered by a mobile FME00 (^Ⓡ^Edibon) Hydraulic Bench equipped with an autonomous tank and pump. Additionally, a centrifugal pump with specifications of 0.37 KW, 30 – 80 L/min, at 20.1 – 12.8 m, single-phase 200 ACV – 240 ACV/50 Hz or 110 ACV – 127 ACV/60 Hz, and a stainless-steel impeller is integrated into the system. The sink tank has a capacity of 165 liters. The hydraulic system is configured with flowmeters and pressure sensors. Upon activating the pump, the system initiates the recording of flows and pressures. The gradual closure of the downstream valve is facilitated through a ½" metal lever ball valve. Four successive closures are executed at intervals of *t* = 5 s, *t* = 10 s, *t* = 15 s, and *t* = 20 s, maintaining a standard flow rate of 12 L/min. The proposed hydraulic prototype was carried out in the Hydraulics Laboratory of the Technological Faculty of the Francisco José de Caldas District University (Bogotá, Colombia). Fig. 1 illustrates the proposed physical prototype, which seamlessly integrates both hydraulic and electronic components. The microcontrollers are supplied with power via the serial ports (5 V). The signals are accessible for monitoring through either the ^Ⓡ^Arduino Oled screens or two ^Ⓡ^ViewBoards. These ^Ⓡ^ViewBoards receive the signal through HDMI and are connected in series. (prototype video ≥≥).

### Prototype

The system comprises two ESP8266 microcontrollers, two USP-G41–1.2 sensors for pressure measurement, two YF-S201 sensors for flow measurement, and two Oled SH1106 monitors. Continuous signals are directed to ports (A0), while pulses are transmitted to pins (D1, D2, D3, and D4). The NodeMCU ESP8266 module receives the signals generated by the sensors from both pin D4 and A0. For flow measurement, the YF-S201 sensor is employed. This sensor is categorized as a turbine-type electronic flowmeter, capturing signals through the magnetic Hall effect. It features three outputs: red (VCC: 5VDC), black (ground), and yellow (pulse output).

**Fig. 2:**
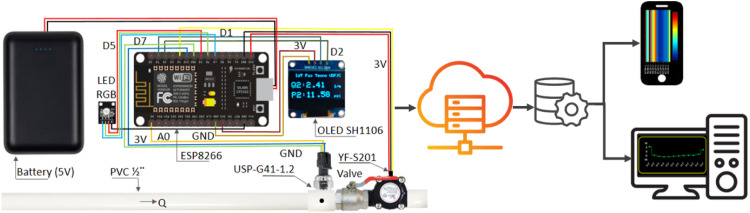
Interconnection block diagram.

The flow enters the sensor activating a magnet in the turbine that has a Hall effect sensor that produces an electrical pulse so that it is possible to count the number of pulses per unit of time generated by the turbine [23] This pulse is transmitted to the ESP8266 microcontroller which sends it via Wi-Fi to the ^Ⓡ^ThingSpeak server. The sensor has a flow range from 1 L/min to 30 L/min, generating average pulses of 2.25 ml and a maximum pressure equal to 1.75 MPa. Likewise, for the measurement of pressure, the USP-G41 sensor was used, which is a pressure transducer that transforms a physical quantity into a voltage proportional to the pressure experienced. Thus, Fig. 2 presents the interconnection block diagram for the hardware and software.

**Fig. 3:**
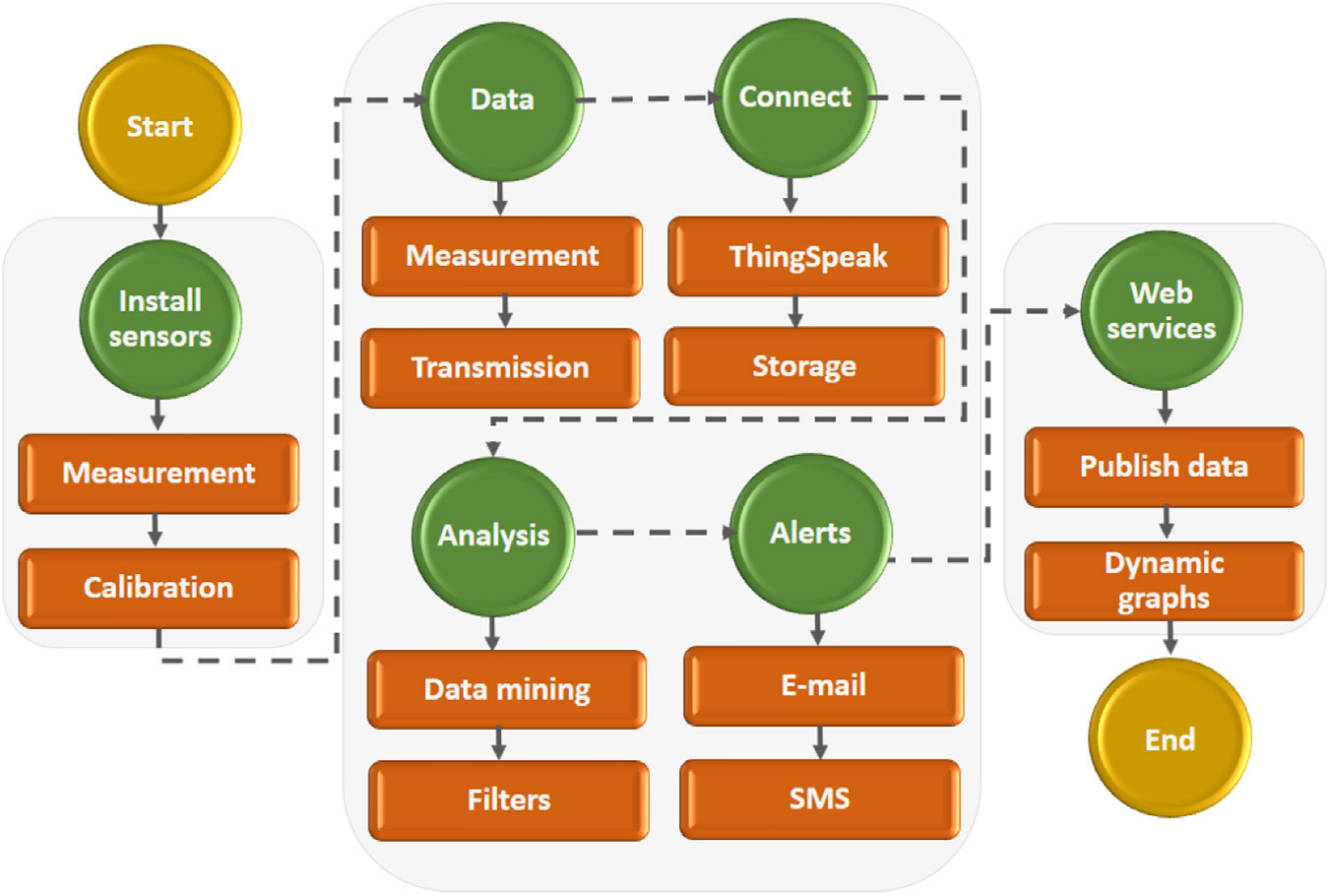
Flow chart measurement process.

The development code under the Arduino platform is open and available through the following link (*download code*), In this code, the calibration of the sensors will be carried out and communication between the Thinkspeak server and the microcontroller will be established. The working current of the sensor is equal to DC 5 *V* ≤ 10 mA with the working voltage of DC 0.5∼4.5 V. The working pressure rate range is 0∼1.2Mpa with a maximum pressure equal to 2.4Mpa and a measurement accuracy of ±1.5 % FS. Finally, the sensor presents a response time of less than 2.0 ms. Fig. 3 shows the flowchart measurement process from sensor installation to web development.

**Fig. 4:**
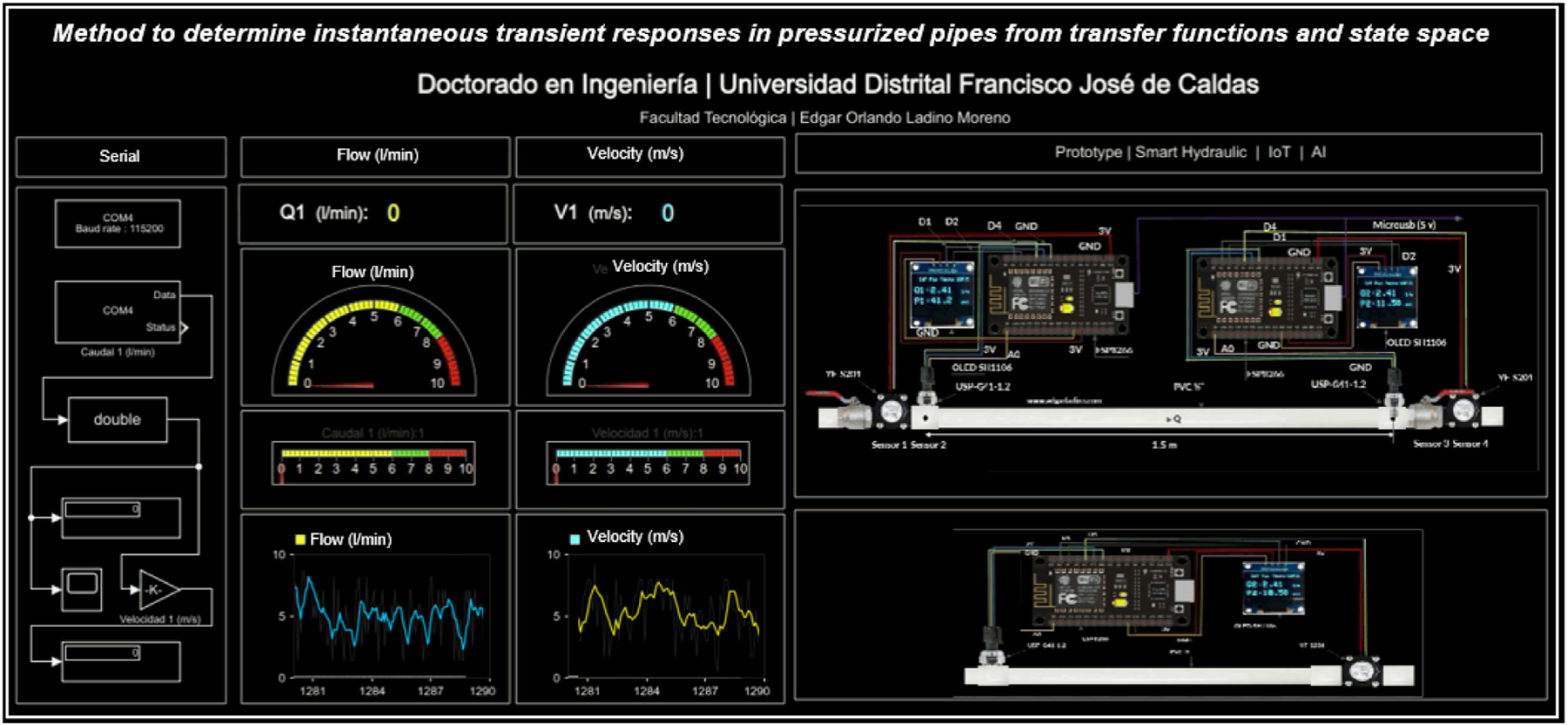
Monitoring dashboard.

The system indicates the behavior of the pressure sensors and the flows downstream and upstream of the system; it also generates graphs of flow and pressure in soft real-time with a 0.1-second delay. This information is sent through the COM3 and COM4 serial ports with a baud rate of 115,200 bauds. The signal transmitted through the COM3 and COM4 serial ports is received in ^Ⓡ^Simulink through the Query instrument block, which configures the arrival of the signal, the port, the type of data received, and the transmission speed. Then, the signal is divided into two parts through a block (transpose). A visualization block (display) and an oscilloscope (scope) are located for monitoring the signal graphically. For the correct operation of data transmission, the serial ports must be previously configured on the computer by entering the device manager. Fig. 4 presents the implementation of a dashboard made in Simulink (^Ⓡ^Matlab) for monitoring the hydraulic system in soft real-time.

### Sensor calibration

**Fig. 5:**
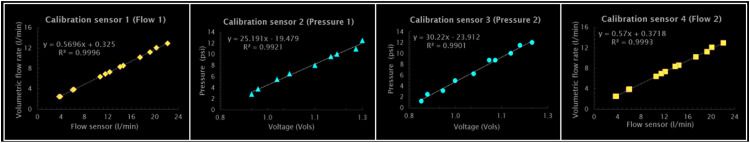
Calibration curves.

The measurement of different physical variables such as flow and pressure in hydraulic systems through sensors constitutes a challenge from the electronic and physical field due to the calibration of the system and the noise inherent in the signal measurement process. The sensor calibration process is essential since the quality of the data depends on it to significantly reduce the random uncertainty of the system. Therefore, it is necessary to establish a calibration standard for each variable. On the one hand, the YF-S201 sensor was calibrated from ten volumetric flow observations for sensors 1 and 4. The accuracy of the sensor is due to the linear relationship between the sensor pulse rate and the flow rate [24] According to the manufacturer's specification, the YF-S201 sensor has a calibration factor (CF) of around 7.5 to 8 with a precision level of 3 - 10 % uncertainty [25] On the other hand, the USP-G41–1.2 pressure sensor was calibrated using a 312.20 analog precision manometer. Ten observations were made for sensors 2 and 3. The signal generated by each sensor was classified according to its fit to the probability density to determine if the signal comes from a stationary or non-stationary random process. The calibration equations presented in Fig. 5 were programmed in ^Ⓡ^Arduino to generate the output value based on the pulse of the input signal for each sensor in soft real-time. The quality of the fit was measured through the correlation coefficient $\left(R^{2}\right)$. For the four sensors, the calibration line obtained a correlation close to unity, which indicates the linear dependence between the signal measured in-situ and the signal captured by each of the sensors.

Likewise, Fig. 5 shows an almost perfect linear relationship between the measurable signals and the sensor responses. The flow sensors (1 and 4) correlate the flow rate in liters per minute with a volumetric output in milliliters, both exhibiting a correlation coefficient greater than 0.999, which denotes high calibration precision. In parallel, the pressure sensors (2 and 3) relate the voltage to the pressure in psi, and both have correlation coefficients greater than 0.99, thus ensuring their reliability.

### Data processing

**Fig. 6:**
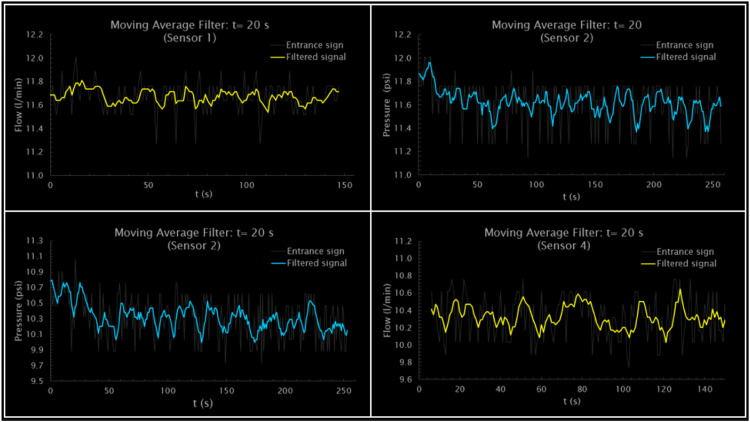
Filtered signals (Mobile media): *t* = 20 s.

The moving average filter (MAF) was implemented, which corresponds to the convolution of the input signal and a rectangular pulse of area 1 introducing a delay to the series. The filter smoothes the input signal produced by the sensors to identify proper patterns in the signal and to minimize existing noise. The adoption of this filter is due to the behavior shown by the series (Fig. 6), the fast step response, and the phase response that presents a linear behavior. For this, the ^Ⓡ^Matlab moving average filter was used, which slides a length window (WindowSize) along the data, calculating the means of the series contained in each window. Eq. (12) defines the filter for a vector x

(12) $$y\left(n\right)=\frac{1}{WindowSize}\left(x\left(n\right)+x\left(n-1\right)+\ldots +x\left(n-WindowSize-1\right)\right))$$

Thus, [26] implements the moving average filter in ^Ⓡ^Matlab and ^Ⓡ^Arduino and shows the oscillograms of the signals both at the input and at the output of the filter. [27] uses ANFIS combined with the ^Ⓡ^Matlab moving average filter to remove disturbances and optimize response time in the preprocessing of large volumes of spectral data. [28] performs a comparison of filters from the data obtained by the MPU-6050 sensor. It also uses a moving average filter, a particle filter, a Kalman filter, and an adaptive Kalman filter. The best results were presented by the adaptive Kalman filter. For example, Fig. 6 presents the filtered signals for a 20-second gradual valve closing time for the four flow and pressure sensors. Sensor 1 (flow) presented the least amount of signal disturbances compared to the measurement of a standard volumetric flow, which obtained a flow of 11.7 L/min. The signal indicates a peak of 12 L/min after 12 s and a minimum value of 11.2 L/min after 45 s. To stabilize the sensors before closing the valve, a period of 140 s was established for the flowmeters and 250 s for the pressure sensors. Likewise, a study of the long-term operation of the prototype has been carried out. The signal has been monitored for four weeks to evaluate its performance continuously and to identify the noise inherent to the prototype and the in situ conditions. Thus, in the event of peaks in terms of pressure, an email is sent generating the alert instantly.

**Fig. 7:**
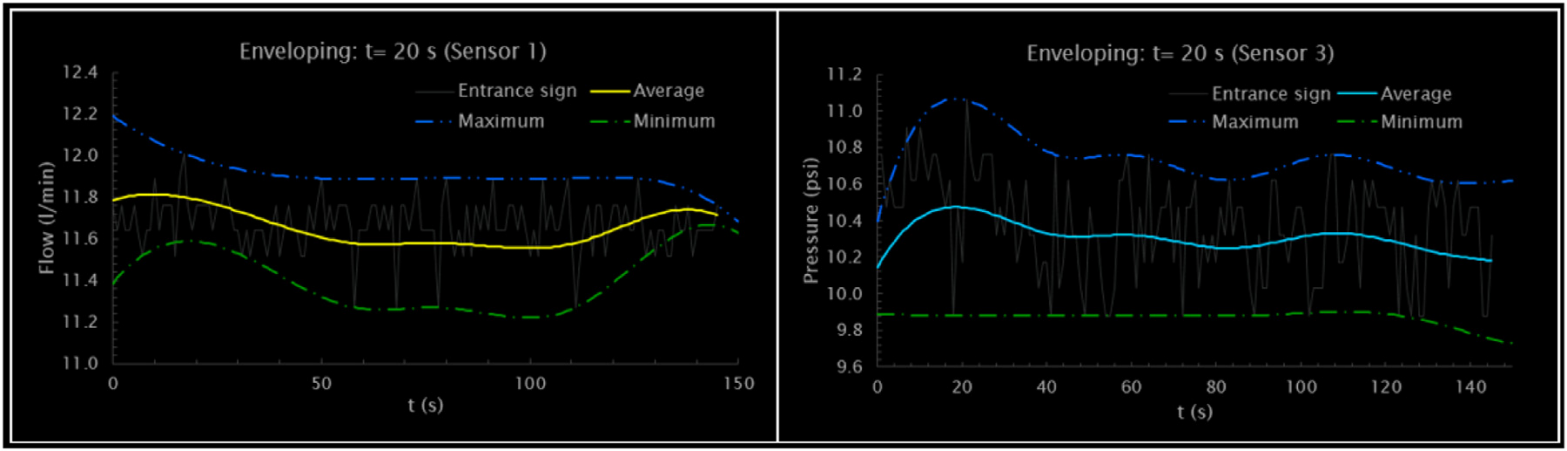
Filtered Flow and pressure enveloping: *t* = 20 s.

Fig. 7 shows the enveloping corresponding to the upper and lower peaks, where the Hilbert transform is applied to extract the enveloping signal. The interpolation process is carried out through splines for the maximum, average, and minimum flow, and pressure. Similarly provided, it shows the application of a moving average filter with a time window of 20 s to the flow and pressure signals recorded by four different sensors. In the graphs of the flow sensors (Sensor 1 and Sensor 4), you can see how the filter smoothes the fluctuations of the input signal, offering a more stabilized line that represents the flow trend. Similarly, for pressure sensors (Sensor 2 and Sensor 3), the filter attenuates rapid variations in the signal, making it easier to identify more significant and long-lasting pressure variations over time.

Fig. 7 shows the transient behavior of the system. The dynamics begin with the opening of the main valve, which significantly affects the behavior of the flow and pressure signal. That is, the hydraulic system presents high sensitivity to the initial conditions due to the turbulence in the system, which indicates the presence of a possible “*strange attractor*”.

**Fig. 8:**
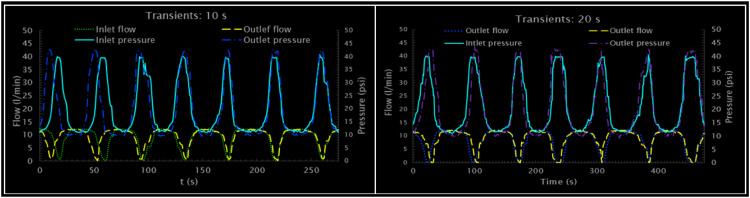
Experimental transients: Flow - Pressure.

Consequently, shows two graphs that apply the envelope technique to the flow and pressure signals for two sensors (Sensor 1 and Sensor 3) with a time window of 20 s. The graph for Sensor 1 reveals the flow signal (in liters per minute) and that of Sensor 3 the pressure signal (in psi). In both cases, three lines are calculated and plotted: the original entry signal, the moving average that represents the average value, and the high and low lines that form the envelope of the signal. The average signal provides a clear view of the central tendency of the sensor's behavior, while the maximum and minimum envelope defines the limits within which the signal varies. Fig. 8 shows the input and output fluctuations of the hydraulic system. Periodic behavior is observed with peaks corresponding to pressure and flow cycles that rise and fall synchronously. The inlet and outlet pressure seem to have a direct relationship with the flow, which indicates a coherent response of the system to transients, evidencing a spatiotemporal variation of flow and pressure.

### Signal modeling

A random signal can be modeled from two functions: an autocorrelation function based on time, and a power spectral density function based on frequencies. The signals generated by the pressure and flow sensors are modeled from a deterministic component (x(t)) associated with a random component or noise (w(t)). For this, how the data is distributed as a function of time must be determined through frequency histograms. For non-stationary signals, the spectral behavior changes as a function of time, which means that for each time instant, there is a different probability distribution. However, the distribution of the probability function for stationary signals remains constant in time. In this way, a stationary process is ergodic if its statistical properties can be determined by a single observation of the series. From the study of the characteristics that the signals present in terms of density functions for the case of non-stationary signals, it is possible to establish a deterministic model added to the random component.

(13) x(t)=μ(t)+w(t)

**Fig. 9:**
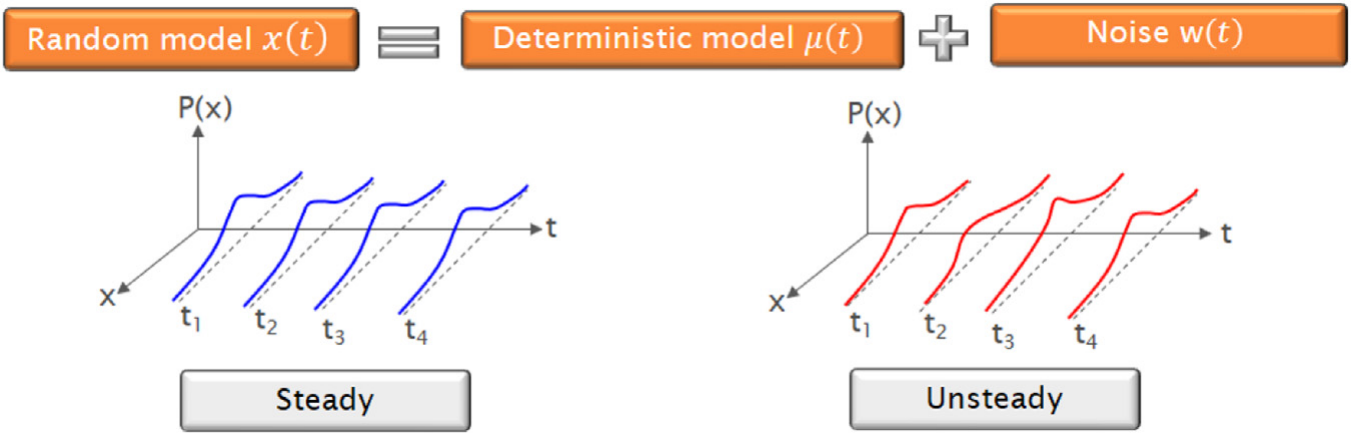
Random models.

The hydraulic variables corresponding to flow and pressure behave randomly, therefore the scheme proposed for mathematical modeling in this study is based on a random model. Fig. 9 presents the behavior of hydraulic signals as a function of time.

**Fig. 10:**
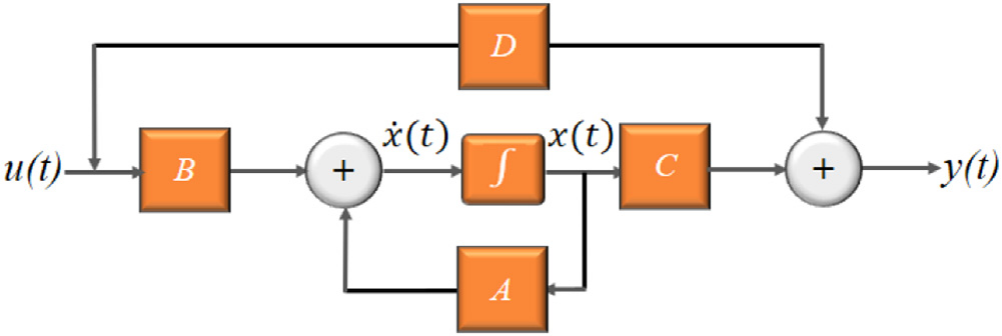
Block diagram for the state-space.

The modeling of the behavior of the transient in the system leads in theoretical terms to the linearization of the partial equations that govern the phenomenon of water hammer, in this case, the non-linear partial differentials of speed and piezometric head. This linearization is obtained by implementing the Laplace transform to generate a transfer function, the algebraic relationship between the output and the input of the system. Likewise, the same system can be modeled under the state space approach in which a dynamic matrix that represents the state of the system is obtained. The fundamental importance of the study of the signals produced by the hydraulic system is to determine the type of function that best fits the signal. For non-stationary signals, it is possible to propose a dynamic transfer function that changes with time and can be calculated in soft real-time. Knowing the type of distribution that governs the signal, it is possible to predict its behavior from the implementation of the adaptive Kalman filter. These filtered data can be the basis for establishing a hierarchical neural model associated with a neuro-fuzzy model to estimate the behavior of the transient under preset boundary conditions before it occurs. Fig. 10 shows the representation of the state-space (flow), where $\dot{x}\left(t\right)$: Dynamic matrix, x(t): states vector, u(t): system input vector, y(t): output vector, A: system matrix (nxn),B: control matrix (mxn), C*:* system matrix (nxn), and D*:* control matrix (mxn). These matrices configure the system coefficients for flow behavior.

## Method validation

### Transitory response: flow

To analyze the pressure and flow data, the ^Ⓡ^Matlab System Identification functions were used in both cases. Nine estimations were employed to fit the data into a system. The estimator functions used were:

• Process model with two poles and delay (P2D)
• Process model with two underdamped poles and delay (P2DU)
• Process Model with two poles, one zero, and delay (P2DZ)
• Process model with two underdamped poles, one zero, and delay (P2DZU)
• Process Model with two poles, one zero (P2Z)
• Process model with two underdamped poles and one zero (P2ZU)
• Transfer function with two poles and one zero (TF2P1Z)
• Transfer function with two poles, one zero, and delay (TF2P1ZD)
• State Space with order 4 (SS4)

All the estimators were chosen to try to follow the physical behavior, considering that the pipe has pressure and flow accumulation, which are two accumulators. Then, we used the ^Ⓡ^Matlab fit percent Normalized Root Mean Squared Error (NRMSE) to measure how well the model fits the estimation data as a percentage gif=100(1−NRMSE). With this information, the three best fits are chosen as possible models for pressure and flow.

### Transitory response: pressure

**Fig. 11:**
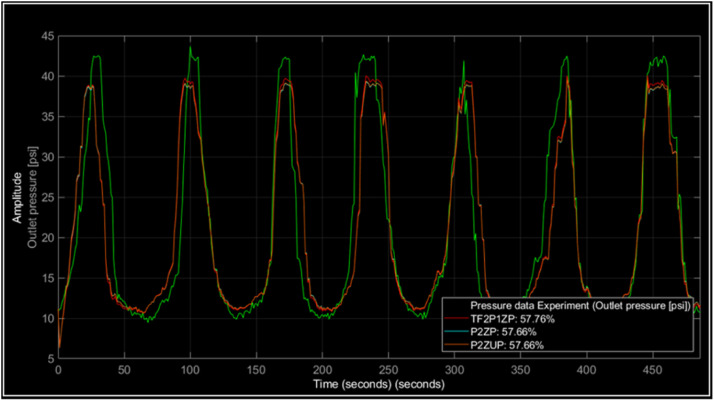
Simulated pressure process vs. experiment.

Using the method described before three models are chosen by the better fits, those models are resumed with the transfer function and fit percent in Table 1. Then the comparison of the three models was made graphically using the experiment data versus the models estimation as can be seen in Fig. 11. It can be seen that the simulation and experimental curves have similar patterns concerning the amplitude and periodicity of the pressure cycles.

**Table 1:** Pressure models.

Model	Transfer function	Fit
TF2P1Z	$G\left(s\right)=\frac{2.9054s+0.2521}{s^{2}+2.8797s+0.2578}$	57.7559%
P2ZU	$G\left(s\right)=\frac{0.9742(8.6288s+1)}{{\left(1.5833s\right)}^{2}+8.6103s+1}$	56.836%
P2Z	$G\left(s\right)=\frac{0.9741(9.0062s+1)}{\left(8.7314s+1)(0.2785s+1\right)}$	56.8356%

**Fig. 12:**
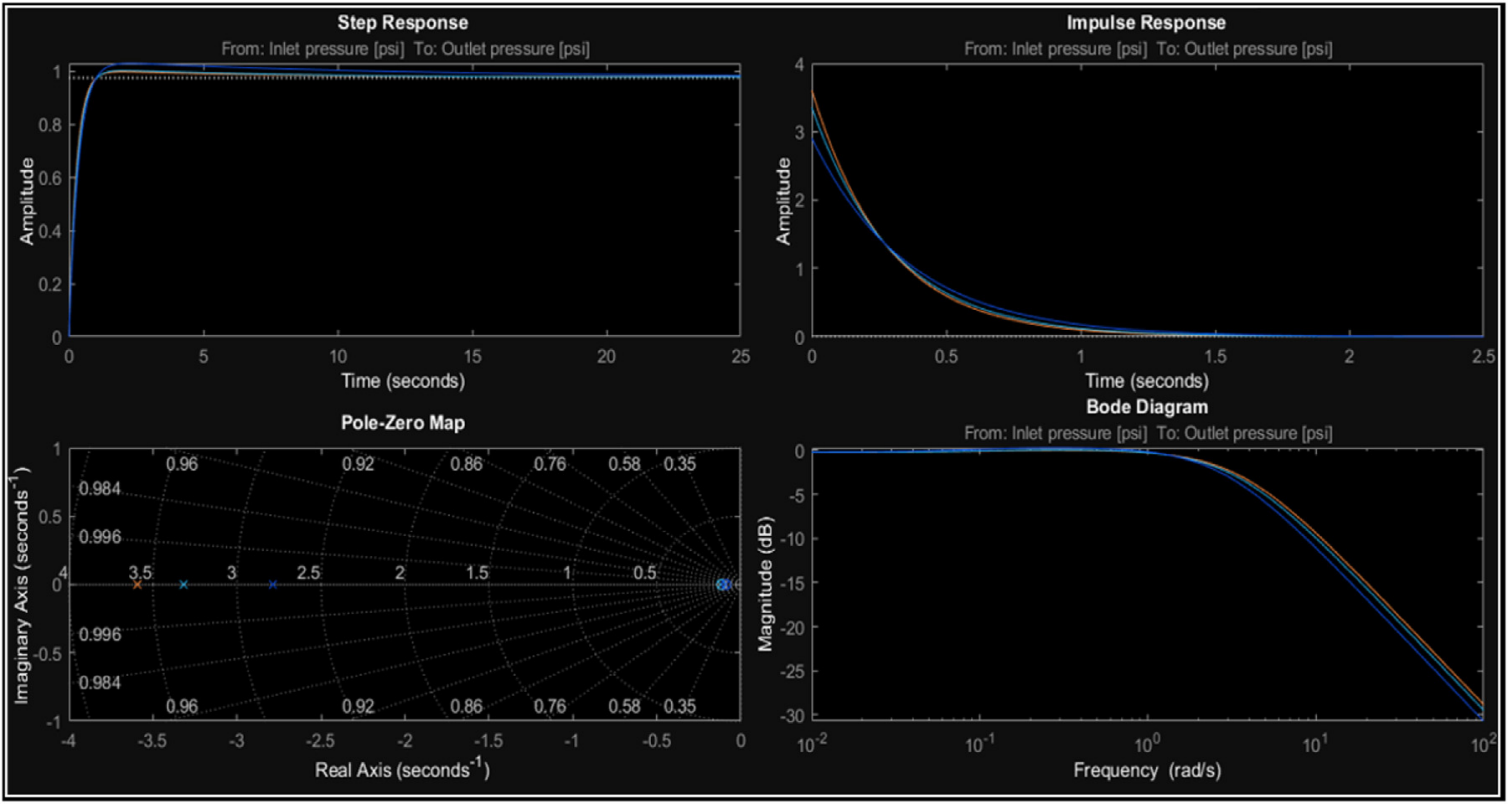
LTI Pressure analysis.

**Fig. 13:**
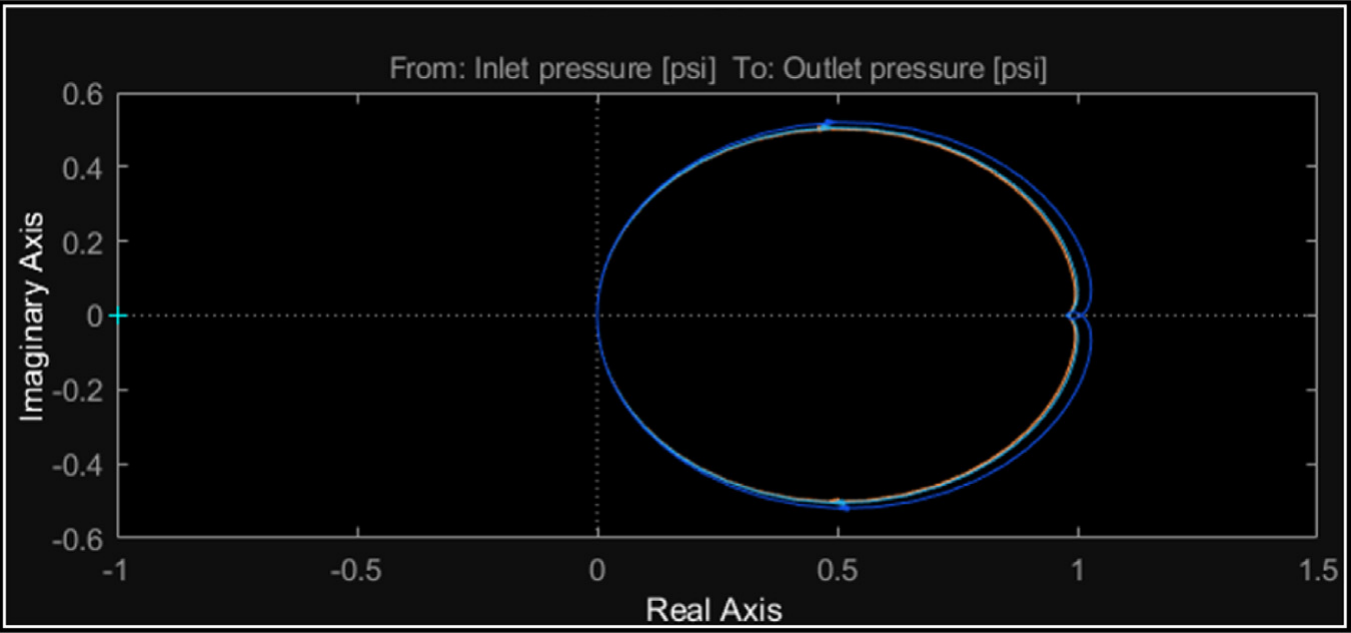
Nyquist diagram.

The response time corresponds to the time it takes the system to reach a specific percentage of its final value after a step input, which is why, for pressure, the step response (Step Response) ranges from 0 to 25 s. Likewise, the Impulse Response amplitude decreases rapidly in approximately 2 s (Fig. 12). In Fig. 13, it can be observed that the models are stable, with all poles located on the left side of the *Pole-Zero Map*. Additionally, the Bode diagram indicates a low-pass behavior, while the Impulse and Step responses hold physical significance in the context of hydraulic processes. In particular, a closer examination of the Nyquist diagram reveals that the system's behavior corresponds to a slight increase in pressure over the reference when the input changes rapidly. Therefore, the trajectory does not enclose the point (−1.0) in the complex plane, which generally indicates that the system is stable according to the Nyquist criterion.

The transient response analysis for flow followed the same method as that for pressure. The three best-fitting simulation process systems were identified using the ^Ⓡ^Matlab tool, and their corresponding transfer functions are summarized below (Table 2).

**Table 2:** Flow models.

Model	Transfer function	Fit
TF2P1Z	$G\left(s\right)=\frac{60.749s+0.0728}{s^{2}+74.2503s+0.0691}$	51.1633%
P2ZU	$G\left(s\right)=\frac{1.0268(38.3765s+1)}{{\left(0.58s\right)}^{2}+46.772s+1}$	50.17%
P2Z	$G\left(s\right)=\frac{1.0281(40.4724s+1)}{\left(48.9129s+1)(0.0266s+1\right)}$	50.1%

**Fig. 14:**
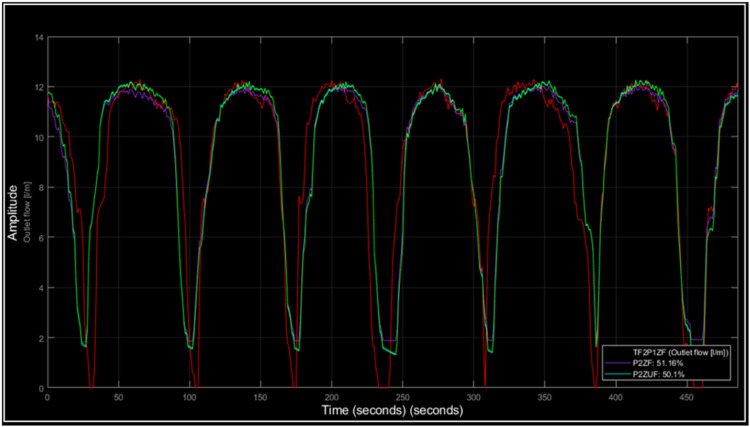
Simulated flow process vs. experiment.

Now the simulations comparing the models with the experiments are observed in Fig. 14. The overlap of the flow curves suggests that the simulation model has a considerable fit with the experimental data, reflected in the calculated fit percentages.

In the graph, it is possible to observe that although the fit percentages are close to 50 %, the simulation accurately captures the experimental behavior. The most notable difference is on the bottom side of the graph, indicating that the model has a slightly higher energy accumulation.

**Fig. 15:**
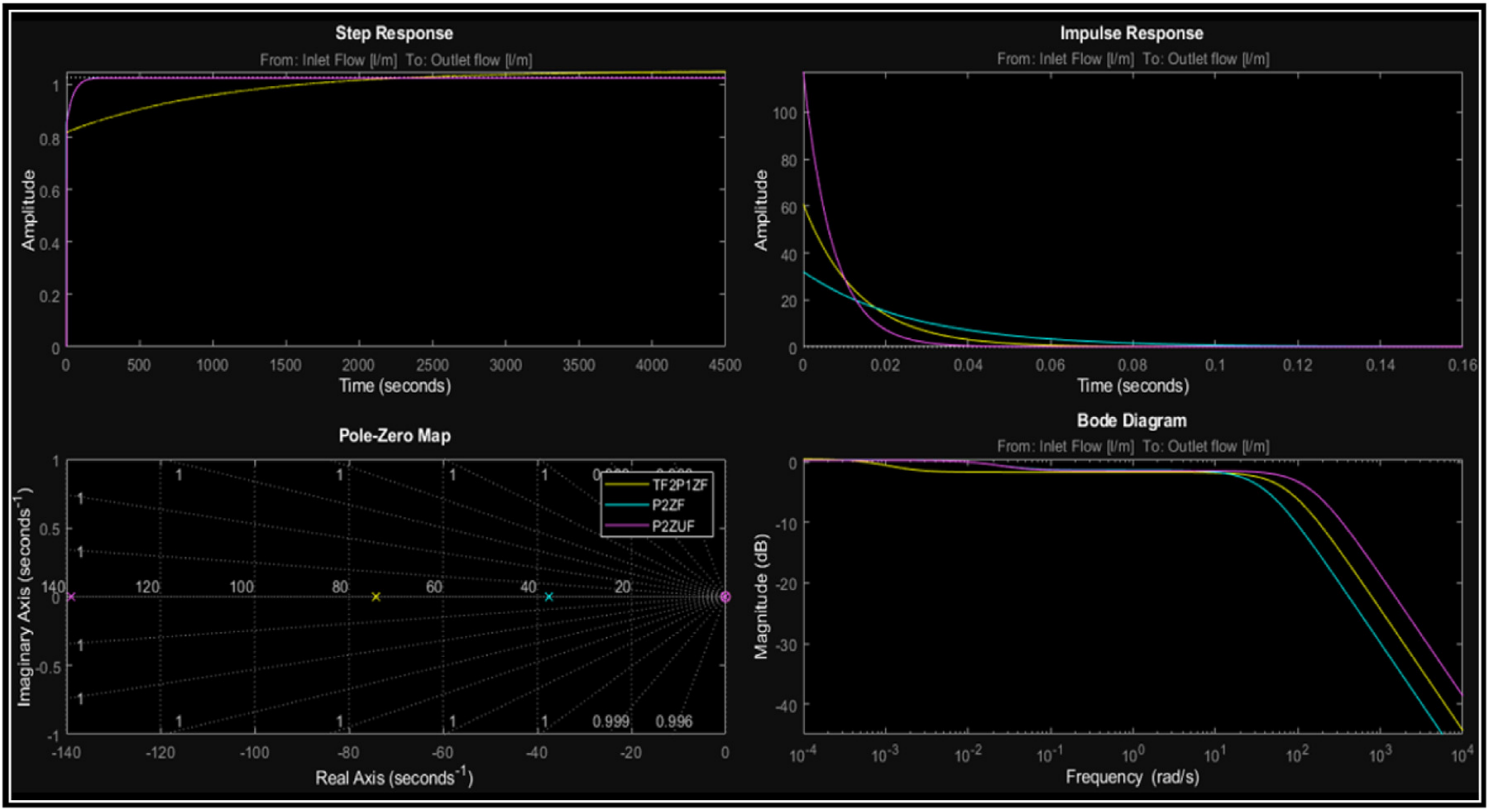
LTI flow analysis.

This is important because this behavior is observed again in the LTI analysis. When the LTI viewer tool was used on these systems, the results are presented in the following (Fig. 15).

The impulse response for the flow signal was 0.16 s, where it is evident how the amplitude of the system changes, starting from a high initial value and decreasing rapidly, which indicates that the system is responding to the disturbance caused by the transient and then the system returns to a steady state. Fig. 15 shows, the step response shows how the system reaches a steady state after a change, evidencing stabilization without oscillations. The impulse response shows that the system reacts and then decays quickly, which is an indicator of stability. In the LTI analysis, it can be observed that all poles are on the left side of the *Pole-Zero Map*, indicating that the systems are stable. Regarding the frequency analysis, the behavior exhibits a low-pass characteristic, signifying that the systems cannot respond to rapid flow changes due to the accumulation of fluid flow in the pipes. Furthermore, the impulse response is consistent with the system. In the case of the step response, the models exhibit minimal overshoot, in line with the physical model, and show a steady-state error relative to the reference. This is consistent with the time analysis behavior but does not significantly affect the potential modeling of the system.

## Conclusions

The prototype developed for real-time transient monitoring in pressurized systems demonstrates the utility of integrating hydraulic systems with IoT technology to establish synchronized early warning networks that alert to overpressure or underpressure events in the system. The integrated use of ^Ⓡ^Matlab and the ^Ⓡ^Arduino platform significantly contributes to understanding the behavior of transients generated by the gradual closing of the valve in a hydraulic system. Thus, the research in this study provides a feasible IoT design and a set of methodological tools for monitoring under transient conditions in pressure pipelines.

When observing the pressure and flow system models versus the experimental behavior, it can be seen that the models closely match the experimental data. Therefore, the models, specifically the transfer functions, can be used to represent the behavior of the systems in various scenarios, such as changes in the frequency of inputs or the introduction of leaks as noise in the outlet part of the system. For example, the root-locus tool can be used to analyze the impact of leaks, and the step and impulse behavior data can be utilized to study hydraulic hammer. This information is valuable for designing system compensators and feedback control to protect hydraulic systems.

Thus, through the methodology of applying transfer functions and state space models, it is possible to evaluate how leaks affect the transient responses of the system. This includes the ability to analyze the magnitude, duration, and frequency of disturbances generated by leaks.

Transient analysis of the proposed prototype reveals that the hydraulic system exhibits high sensitivity to initial conditions due to turbulence produced in the system. This suggests the presence of a potential *dynamic strange attractor* for phenomena caused by water hammer in pressure pipes. In terms of flow and pressure, the hydraulic system is stable because the zeros and poles of the transfer functions are located on the left side of the complex s-plane. Finally, in future studies, it will be possible to monitor the transient behavior of leaks through IoT and artificial intelligence techniques that incorporate machine learning for the location and quantification of leaks, proposing a *dynamic transfer function* that changes instantly over time.

## Limitations

None

## Ethics statements

The method discussed in this scientific article did not involve studies with living beings.

## CRediT author statement

**Edgar Orlando Ladino-Moreno**: Prototype, Research, Validation, Methodology, Supervision, Software, Code, Programming, Formal Analysis, Writing-Review and Editing. **César Augusto García-Ubaque:** Methodology, Conceptualization, Data Curation, Formal Analysis, Writing-Revision, and Editing. **Oscar Gabriel Espejo-Mojica:** Validation, Methodology, Supervision, Software, Code, Programming, Formal analysis, Writing-Review and Editing.

## Declaration of interests

The authors declare that they have no known competing financial interests or personal relationships that could have appeared to influence the work reported in this paper.

## Data Availability

Data will be made available on request.
